# Supplementary material for: Vitamin D Modified DSS-Induced Colitis in Mice via STING Signaling Pathway
Source: Biology (Basel). 2025 Jun 18;14(6):715. doi: 10.3390/biology14060715 (PMC12190092; doi:10.3390/biology14060715)
Supplement: Supplementary file 1 [file biology-14-00715-s001.zip › Supplementary Materials S1: 16s-report of gut microbiota/Groups/diff_analysis/Correlation/Genus-METACYC.pdf]

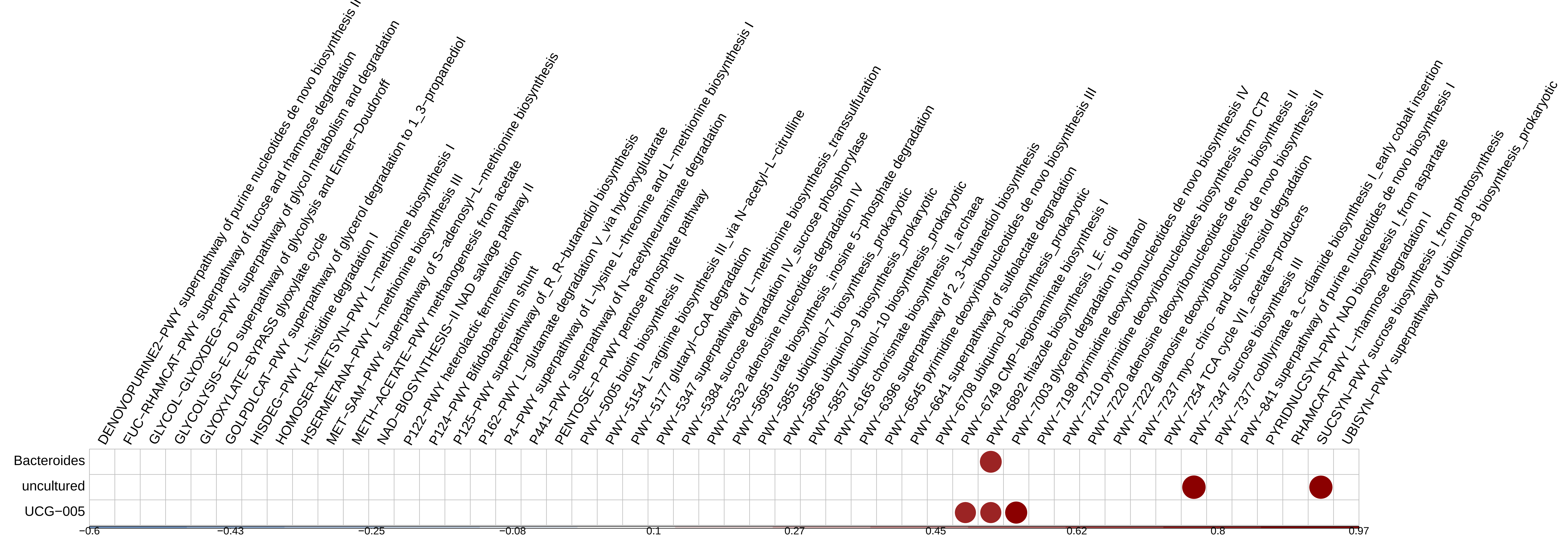
DENOVOPURINE2−PWY superpathway of purine nucleotides de novo biosynthesis II
FUC−RHAMCAT−PWY superpathway of fucose and rhamnose degradation
GLYCOL−GLYOXDEG−PWY superpathway of glycol metabolism and degradation
GLYCOLYSIS−E−D superpathway of glycolysis and Entner−Doudoroff
GLYOXYLATE−BYPASS glyoxylate cycle
GOLPDLCAT−PWY superpathway of glycerol degradation to 1_3−propanediol
HISDEG−PWY L−histidine degradation I
HOMOSER−METSYN−PWY L−methionine biosynthesis I
HSERMETANA−PWY L−methionine biosynthesis III
MET−SAM−PWY superpathway of S−adenosyl−L−methionine biosynthesis
METH−ACETATE−PWY methanogenesis from acetate
NAD−BIOSYNTHESIS−II NAD salvage pathway II
P122−PWY heterolactic fermentation
P124−PWY Bifidobacterium shunt
P125−PWY superpathway of_R_R−butanediol biosynthesis
P162−PWY L−glutamate degradation V_via hydroxyglutarate
P4−PWY superpathway of L−lysine L−threonine and L−methionine biosynthesis I
P441−PWY superpathway of N−acetylneuraminate degradation
PENTOSE−P−PWY pentose phosphate pathway
PWY−5005 biotin biosynthesis II
PWY−5154 L−arginine biosynthesis III_via N−acetyl−L−citrulline
PWY−5177 glutaryl−CoA degradation
PWY−5347 superpathway of L−methionine biosynthesis_transsulfuration
PWY−5384 sucrose degradation IV_sucrose phosphorylase
PWY−5532 adenosine nucleotides degradation IV
PWY−5695 urate biosynthesis_inosine 5−phosphate degradation
PWY−5855 ubiquinol−7 biosynthesis_prokaryotic
PWY−5856 ubiquinol−9 biosynthesis_prokaryotic
PWY−5857 ubiquinol−10 biosynthesis_prokaryotic
PWY−6165 chorismate biosynthesis II_archaea
PWY−6396 superpathway of 2_3−butanediol biosynthesis
PWY−6545 pyrimidine deoxyribonucleotides de novo biosynthesis III
PWY−6641 superpathway of sulfolactate degradation
PWY−6708 ubiquinol−8 biosynthesis_prokaryotic
PWY−6749 CMP−legionaminate biosynthesis I
PWY−6892 thiazole biosynthesis I_E. coli
PWY−7003 glycerol degradation to butanol
PWY−7198 pyrimidine deoxyribonucleotides de novo biosynthesis IV
PWY−7210 pyrimidine deoxyribonucleotides biosynthesis from CTP
PWY−7220 adenosine deoxyribonucleotides de novo biosynthesis II
PWY−7222 guanosine deoxyribonucleotides de novo biosynthesis II
PWY−7237 myo− chiro− and scillo−inositol degradation
PWY−7254 TCA cycle VII_acetate−producers
PWY−7347 sucrose biosynthesis III
PWY−7377 cobIIyrinate a_c−diamide biosynthesis I_early cobalt insertion
PWY−841 superpathway of purine nucleotides de novo biosynthesis I
PYRIDNUCSYN−PWY NAD biosynthesis I_from aspartate
RHAMCAT−PWY L−rhamnose degradation I
SUCSYN−PWY sucrose biosynthesis I_from photosynthesis
UBISYN−PWY superpathway of ubiquinol−8 biosynthesis_prokaryotic
Bacteroides
uncultured
UCG−005
−0.6
−0.43
−0.25
−0.08
0.1
0.27
0.45
0.62
0.8
0.97
